# 257. Coinfection Characteristics among Children with COVID-19

**DOI:** 10.1093/ofid/ofac492.335

**Published:** 2022-12-15

**Authors:** Itzel Villanueva García, Nancy Evelyn Aguilar Gómez, Aarón Espinosa Atri, Angela Patricia Vedia Márquez, Juan Antonio Gallegos Marin, Ana Ruth Hernández Tepach

**Affiliations:** Instituto Nacional de Pediatria, Mexico, Distrito Federal, Mexico; Instituto Nacional de Pediatria, Mexico, Distrito Federal, Mexico; Instituto Nacional de Pediatría, Mexico, Distrito Federal, Mexico; Instituto Nacional de Pediatría, Mexico, Distrito Federal, Mexico; Instituto Nacional de Pediatria, Mexico, Distrito Federal, Mexico; Instituto Nacional de Pediatria, Mexico, Distrito Federal, Mexico

## Abstract

**Background:**

Coinfection in patients with SARS-CoV-2 has been associated with greater complications. We describe the clinical characteristics and outcomes of 126 pediatric patients with COVID-19 and viral, bacterial, or fungal coinfection.

**Methods:**

We retrospectively reviewed and analyzed electronic data of all pediatric patients who tested positive for SARS-CoV-2 from April 16, 2020, to April 15, 2022, in our center. Confirmation of COVID-19 was based on positive RT-PCR. Viral coinfections (VC) were identified using a multiplex RT-PCR respiratory viral panel, bacterial coinfection (BC) was determined by positive bacterial culture (blood, bronchoalveolar lavage, sputum, urine) or clinical/radiological manifestations and antimicrobial assessment by a pediatric Infectious Diseases expert and fungal coinfection (FC) diagnosis based on Consensus definitions of invasive fungal disease.

**Results:**

During the study period, among 400 pediatric patients with COVID-19, 126 children had coinfection. Children >10 years were the most affected age group. Underlying disease was present in 69%, hematological malignancies were the most common (17.5%). BC was detected in 76.9% (n=97), bacterial pneumonia (54.6%) was the main diagnosis, followed by oncologic patients with initial febrile neutropenia and posterior SARS-CoV-2 detection (14,4%). Unusual BC as congenital syphilis w detected; acute appendicitis was the initial presentation of COVID-19 in 8 patients. VC was identified in 15.87% (n=20), prevailing rhinovirus (9.5%) and adenovirus (3.96%), One FC presented as proven pulmonary aspergillosis (0.8%). B-V and B-F coinfection were detected in 2 patients. Fever and cough were the most common symptoms, higher fever >40°C was mostly observed in the BC group (3%). Twenty-seven patients with BC (27.8%) were admitted to intensive care, with the OR 0.7 IR 95% (0.611–1.008), 4.1% died. One ICU admission was observed in the VC group (5%) and all VC cases resolved without complications.

**Conclusion:**

Pediatric patients with COVID-19 coinfection, especially BC were common in our center representing nearly one-third of the infected children, including unusual coinfections. BC was identified as a risk factor for ICU admission OR 0.7 IR 95% (0.611–1.008). Favorable outcomes were observed in most cases.

**Disclosures:**

**All Authors**: No reported disclosures.

